# Maternal Trophic Status and Offpsring Phenotype in a Marine Invertebrate

**DOI:** 10.1038/s41598-018-27709-2

**Published:** 2018-06-25

**Authors:** Enrique González-Ortegón, Lewis Le Vay, Mark Edward Mackay Walton, Luis Giménez

**Affiliations:** 1 Instituto de Ciencias Marinas de Andalucía (ICMAN-CSIC), Campus Universitario Río San Pedro, 11519 Puerto Real, Cádiz Spain; 2 School of Ocean Sciences, Bangor University, Menai Bridge, LL59 5AB UK; 3 CEI-MAR International Campus of Excellence of the Sea, Cádiz, Spain

## Abstract

Offspring size variation in relation to maternal size and season is characteristic of a range of species living in seasonal environments. Little is known about the proximate mechanisms explaining the links between maternally driven variation in offspring phenotypes, for instance when mothers have different diets depending on their size or the season. Here, we use stable isotopes techniques to quantify size dependent and seasonal variations in diet in mothers of shrimp *Palaemon serratus* and explore possible links between maternal diet and phenotype of embryos and freshly hatched larvae. We found that larger females, which occur more frequently in winter, produce larvae with higher carbon and nitrogen content as well as higher percent carbon, than smaller mothers collected in winter. In addition, isotopic composition suggest that larger mothers collected in winter, were feeding at a higher trophic level, or on an enriched prey pool compared with smaller mothers collected in summer. Overall, there seems to be a strong association between offspring size and maternal diet, mediated by maternal size and/or season.

## Introduction

In many animals and plants the maternal phenotype and environment are two of the most important determinants of egg size, offspring reserves, performance and survival^1–3^. Egg size or mass usually correlates positively with offspring performance and fitness, a phenomenon known as “the bigger the better”^4^. Intraspecific variation in offspring size found in nature has been associated to the maternal phenotype or the environment experienced by mothers. For instance, there are a large number of cases where offspring size or reserves correlates with female size^5–8^, or where for instance offspring traits fluctuate seasonally^2,9^. The mechanisms behind such a patterns are not well resolved. Several adaptive explanations are available for the case of maternal-offspring size correlations^7^, including the hypotheses stating that the maternal size may be a proxy for the quality of the offspring environment or that the correlation arises if offspring size drives the overhead costs incurred by mothers during reproduction^10^. Seasonal variations in offspring size for instance are usually attributed to effects of temperature on development^11^ or adaptive responses to the offspring food environment^9^.

Maternal influences on offspring reserves must be based on some behavioural-physiological mechanism. For example, variations in offspring reserves can occur because mothers of different sizes may select different prey or because they access different prey pool, if e.g. females produce offspring in different seasons. Variations associated to environmental factors may be driven by either changes in diet or changes in allocation of reserves at the time of oogenesis. Maternal diet can affect important traits such as gonadosomatic index^12^ and offspring size^2,13^. The maternal diet, in particular, is a major contributor towards chemical composition of eggs^14,15^. In addition, the fact that individual variation in diet within a population is common^16,17^ suggests that diet may lead to intraspecific variation in egg size and offspring reserves.

Here, we studied relationships between seasonal variation in maternal diet and offspring traits for the shrimp *Palaemon serratus*, a marine invertebrate with dispersive larval stages. This is the starting point to determine whether diet underpins natural variation in offspring reserves. Such observation will then motivate further experimental approaches to determine whether associations are driven by cause-effect relationships. In contrast, the lack of variability in diet or the lack of association among offspring size and maternal diet may help to orient research into alternative directions. *Palaemon serratus* inhabits (seasonal) coastal areas of North Europe and develops through a larval phase characterised by a large, but variable, number of stages^18,19^. Large females of *P. serratus* produce larvae with higher dry mass and higher capacity to tolerate food limitation^19^. Females produce eggs, mostly in winter^20^ and larvae hatch in May^18^. However, in late spring, proportionally smaller females are still found carrying early stage embryos. Hence, at the time of egg production, large and small females may differ in their diet because their body size limits the maximum prey size or because of seasonal variations in the quality of prey. Offspring traits were evaluated as dry mass, carbon and nitrogen content; carbon and nitrogen are proxies for lipids and proteins respectively^21^. Maternal diet was evaluated from isotopic composition (N and C) of female tissue, embryos and larvae. In palaemonid shrimps, the process of allocation of reserves into eggs, vitellogenesis, takes place over a long time period: primary vitellogenesis occurs over several moults and secondary vitellogenesis from one to three molt cycles^22,23^. Hence, as stable isotopes reflect the assimilated diet over a longer time period^24,25^, they are more useful than the dietary information provided by analysis of stomach content at the time when females carry their eggs^26^.

Isotopes of N and C have been used to ascertain relationships between consumers and their diet^27–29^. Isotopic shifts with ontogenetic development or changes in body size can be the result of feeding on isotopically different food during the growth period, mostly through incorporation of dietary isotopes in new tissue growth plus metabolic turnover in existing tissue^30,31^. Temperature is another important source of variability of trophic fractionation in temperate ecosystems^32^. For instance, seasonal temperature variation in δ^15^N signal of prey may cause corresponding variation in muscle of predators or negative relationships between δ^13^C and δ^15^N in zooplankton species has been found across a temperature gradient^33^. Furthermore, the ratio of stable isotopes of nitrogen (δ^15^N) is used to estimate trophic position of a consumer in a food web; as the δ^15^N of a consumer is typically enriched by 3–4‰ as compared to its diet^34,35^. Importantly, trophic position is typically expected to increase with growth or body size^36,37^ but see^38,39^. Isotopic composition is known to vary in response to body size^34,35^ and season due to seasonal variations in e.g. food availability or food selection^40^. We hypothesised a positive relationship between female body size and δ^15^N, based on the previous studies evaluating size-dependent variability in trophic position^34,35^. In addition, stable isotope techniques may be used as a way to infer differential allocation of biochemical reserves into offspring^41^. For example, compared to carbohydrates and proteins, lipids are known to be depleted in ^13^C; hence the proportion of lipids may influence overall δ^13^C^42^. However, there are relatively few studies applying stable isotopes to investigation of the effects of maternal nutrition on offspring quality^43^. We also hypothesised that isotope values in eggs and larvae would reflect maternal diet; such differences may point towards differential transfer of essential amino acids or changes in proportion of lipids allocated as lipo-glyco-protein reserves. Understanding these sources of variation will improve the interpretation of stable isotope analysis and make important steps towards establishing the contribution of maternal nutrition to offspring performance.

## Results

### Elemental carbon and nitrogen composition in females, embryos and larvae

**Figure 1:**
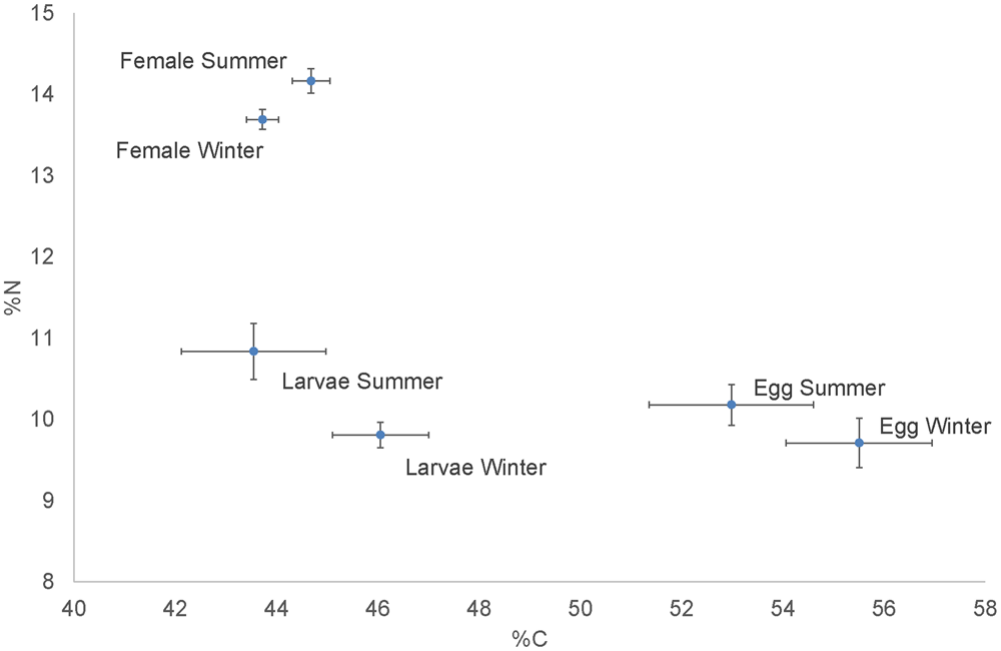
Mean and standard deviation of the carbon and nitrogen content for eggs, larvae and female muscles sampled over 2 seasons (winter vs. summer).

**Figure 2:**
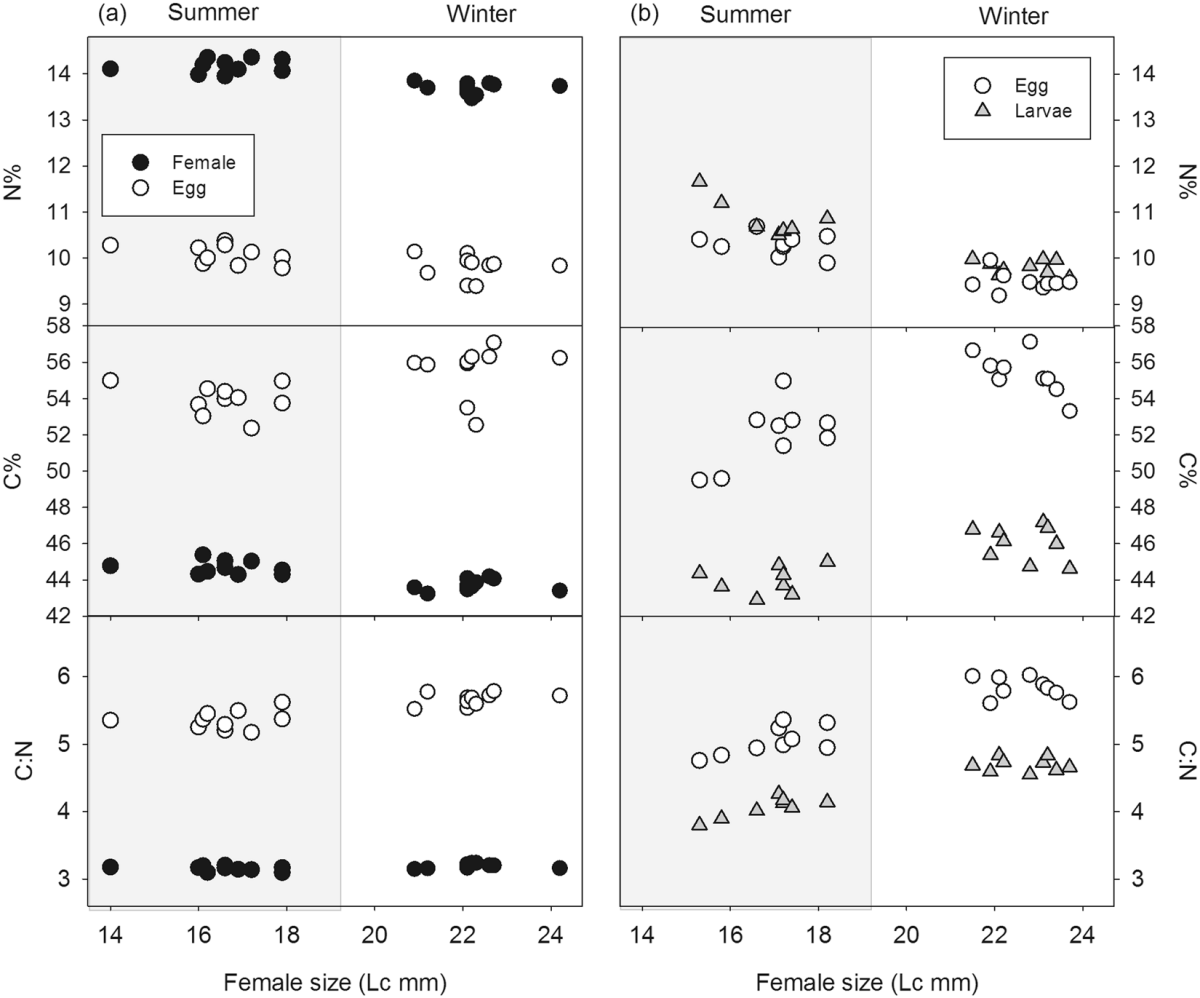
Relationships between female body size and the carbon and nitrogen percentage and the C/N ratio for the links females and eggs (**a**) and eggs and larvae (**b**) in summer and winter. Data of the link eggs-larvae (**b**) correspond to the second batch of females.

The carbon and nitrogen content and C/N differed significantly between season and stage with effects of stage sometimes depending on season (Supplementary Table S1: Model 1). However, the strongest effects were seen between stages; as expected from differences in tissue composition, female muscle had significantly higher %N and lower C/N than eggs and freshly hatched larvae (Fig. 1 and Supplementary Fig. S1). In addition, eggs had higher %C than larvae. Effects of season consisted mainly in increased %C and decreased %N in winter than in summer, especially in eggs and larvae (Fig. 2).

Model 2 (Supplementary Table S1) showed that larger females, found in winter, produced eggs and larvae with higher %C and lower %N (Fig. 2). Significant interactions denoted slight changes in these general trends. Model 3 (Supplementary Table S1) showed that linear effects of female size accounted for effects of season found in model 1; interactions between season and female size, found in most response variables must reflect variation that is not accounted for by the linear effects of female size.

When we compared data separated by season (Table S1: Model 4), we found that females and early egg traits did not show any significant trend with female size. However, the data set considering egg and larval traits did show a significant trend especially in summer (Table S1: Model 4.2), with larger females producing eggs with higher C:N, higher %C and lower %N (Fig. 2); for %C, this trend was stronger in eggs than in larvae (significant interaction St × Lc).

**Figure 3:**
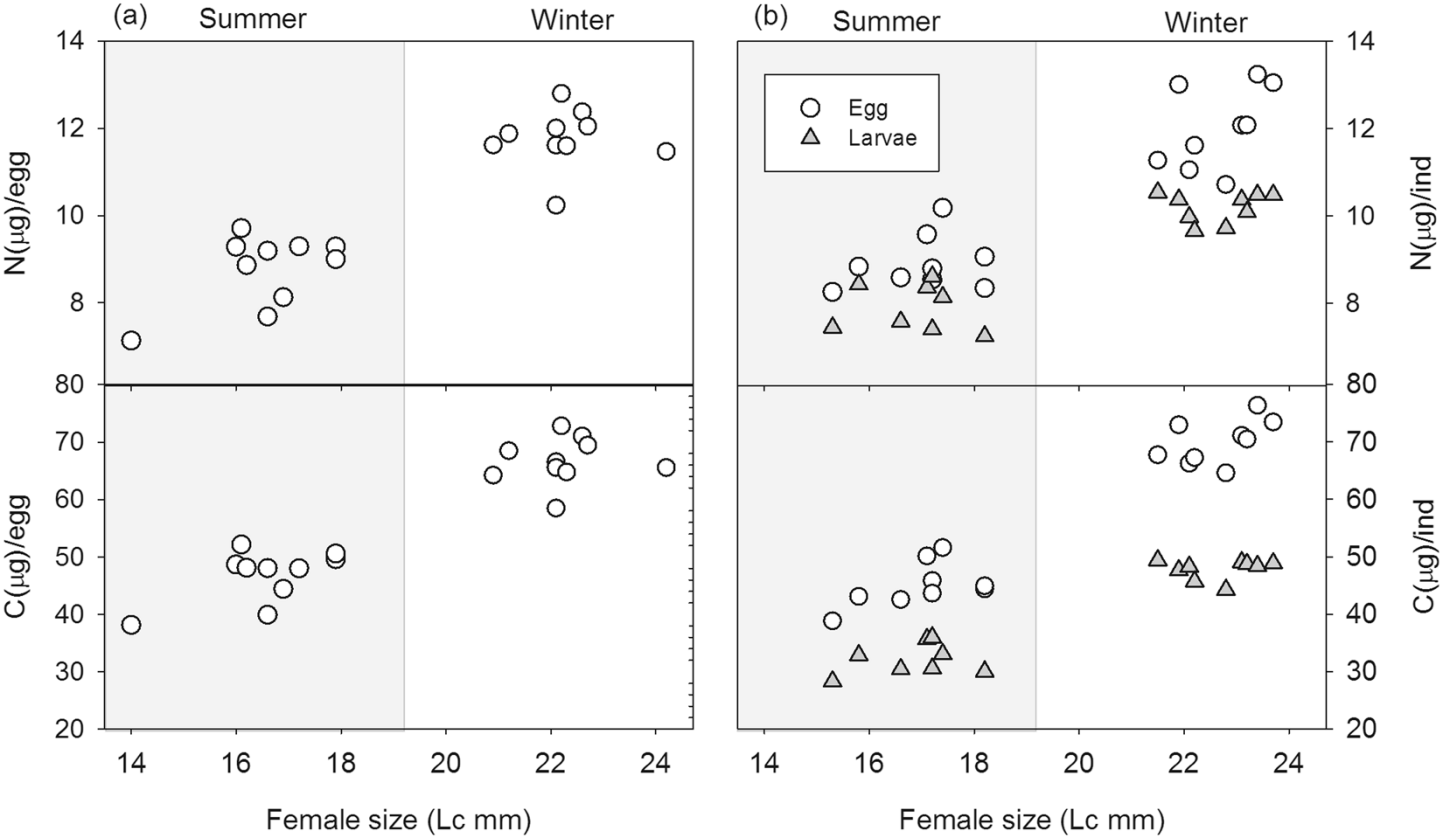
Relationships between female body size and the total carbon and nitrogen content for eggs and larvae per individual from the samples female-eggs (**a**) and eggs-larvae (**b**) in summer and winter. Data of the link eggs-larvae (**b**) correspond to the second batch of females.

Total carbon and nitrogen per egg and larvae were significantly higher in winter (Model 1) and increased with female size (Model 2, Fig. 3). Comparisons of total carbon and nitrogen per larvae and eggs shows a stronger effect in eggs (Fig. 3), suggesting that mainly carbon but also nitrogen were lost during embryogenesis, especially in winter embryos produced by large females.

### Isotopic composition of females, eggs and larvae

**Figure 4:**
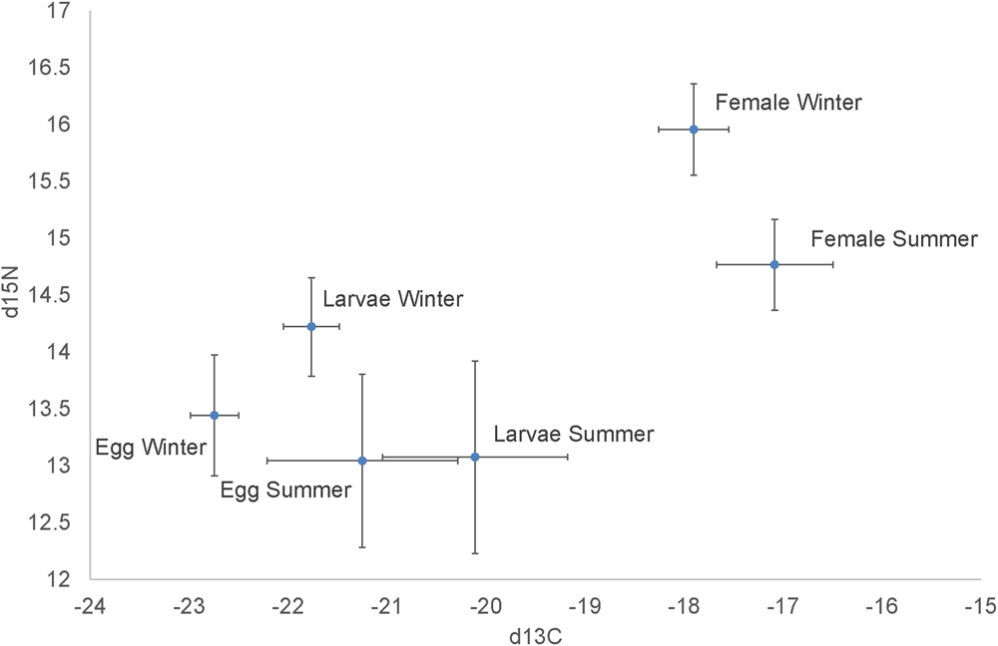
Mean and standard deviation of the carbon and nitrogen isotopic signal (‰) of the eggs, freshly hatched larvae and female muscles sampled over 2 seasons (winter vs. summer).

The δ^13^C and δ^15^N isotopic values showed significant differences between seasons (Model 1: Supplementary Table S1 and S2) with winter females showing lower δ^13^C and higher δ^15^N than summer females (Fig. 4); similar differences were found in eggs and larvae, reflecting the differences in maternal nutrition (Fig. 4). In addition, there were significant differences in isotopic composition between mothers and eggs; these are not further considered here because they are expected from the different tissue types: maternal samples consisted in muscle tissue while offspring samples included lipid reserves, muscle and other tissues.

**Figure 5:**
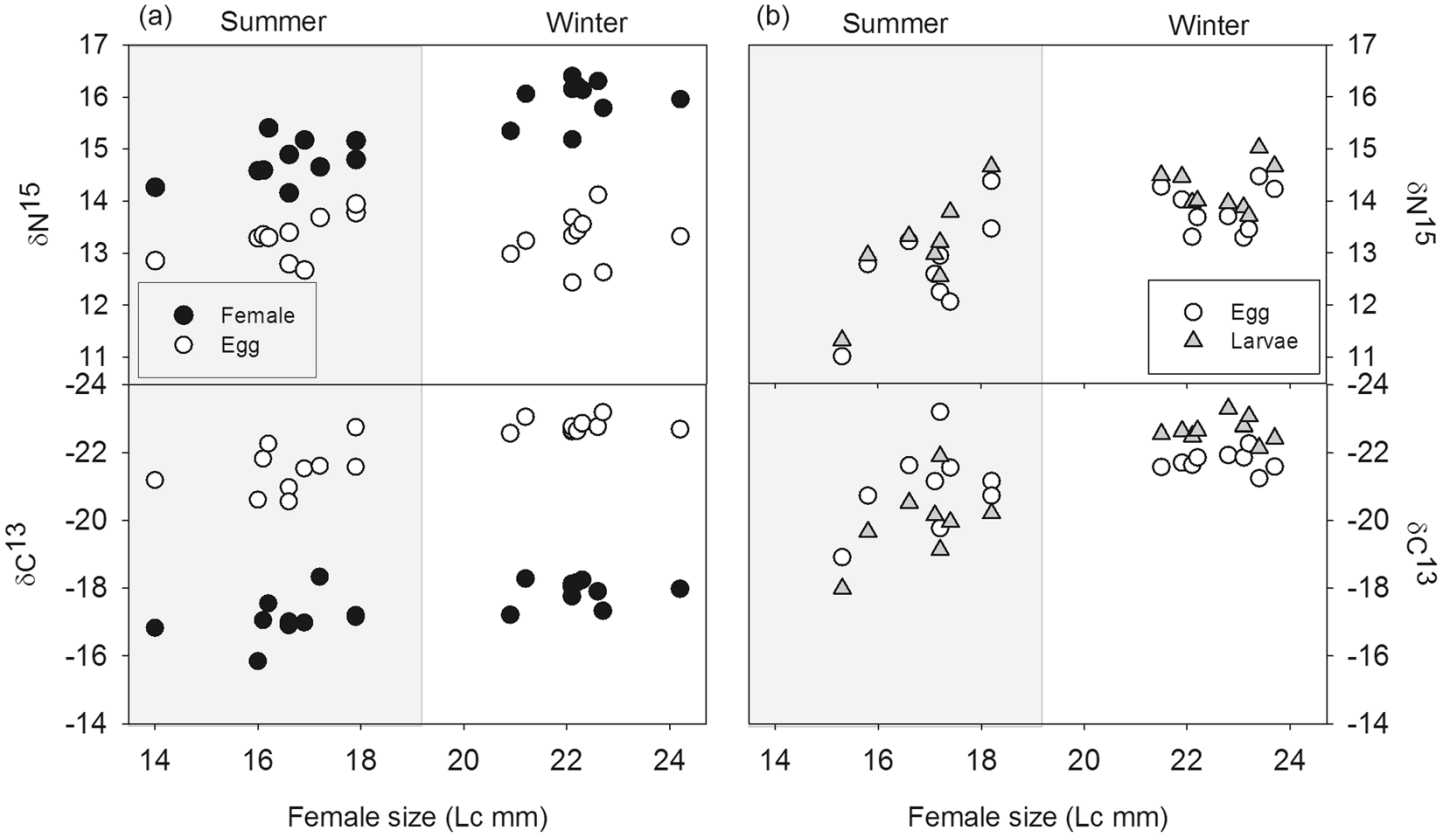
Relationships between female body size and the carbon and nitrogen isotopic signal (‰) for the links between females and eggs (**a**) and eggs and larvae (**b**) in summer and winter. Data of the link between eggs-larvae (**b**) correspond to the second batch of females. Note that in the lower panels the scale is reversed so that δ^13^C increases downward.

Model 2 showed that both δ^13^C and δ^15^N isotopic values increased significantly with female body size (Supplementary Table S2) in mothers, eggs and larvae (Fig. 5). In the sequential tests (Model 3), the factor season was not significant once the variation explained by female size was taken into account.

When we analysed the response of δ^13^C and δ^15^N isotopic values to female body size, but separated by season (Models 4.1 and 4.2: Supplementary Tables S1 and S2), we found a positive correlation between isotopic values and female size for the summer cohort but not for the winter cohort. Averages and coefficients of variation in δ^13^C and δ^15^N are shown in Supplementary Table S3. Coefficients of variation were larger in summer than in winter, for females, larvae and eggs.

## Discussion

We found strong correlations between natural variation in per offspring investment, offspring quality (e.g. %C), female body size and maternal diet (as inferred from isotopic composition) in the shrimp *Palaemon serratus*. Egg and larvae of shrimp *Palaemon serratus* had higher average carbon and nitrogen content produced by larger females extruding eggs in winter, in comparison to smaller summer females. Winter females allocated a slightly higher proportion of carbon and possibly a higher proportion of lipid reserves. In addition, egg and larval isotopic values highlighted the maternal influence on offspring tissues: both females, embryos and larvae had in winter a higher d^15^N and lower d^13^C than in summer.

We cannot separate potential effects of seasonal variation in the quality and amount of food available to females from the effects of female body size on food choices on traits or isotopic values; our findings do not imply cause-effect relationship between diet and offspring traits. We therefore can only speculate on the role of season or females size. For instance, evidence based on other marine organisms would support the hypothesis that season, not body size, may drive offspring traits. In another north European species, *C. crangon*, seasonal variations in offspring size are not sustained by concomitant variations in female size^9^. If diet were the driver of offspring investment, perhaps, seasonal variations in the diets enable the concomitant variation in offspring investment rather than maternal size. One would be tempted to conclude that differences in diet of *P. serratus*, if any, would be driven by seasonal patterns of prey quality rather than maternal size. Isotopic values are known to vary seasonally in pelagic organisms^32,40^ indicating a higher trophic position in winter than in summer as shown by our data. Given the important seasonal changes in the abundance and distribution of prey such as planktonic organisms in temperate habitats, it should be expected that diet varies seasonally^44^. Hence, winter females may feed on a higher trophic level than summer females. This would explain, the variability of the ^13^C and ^15^N isotopic values in female muscle: low variation in winter may reflect expected low prey diversity at that time as compared with summer. Alternatively, isotopic values may reflect seasonal differences in the isotopic composition of primary producers^45–47^ and hence variation in the isotopic signatures of the prey of the shrimp^48^. Evidence against the first alternative hypothesis is supported by the group of winter females, feeding all in the same habitat, showing low variability and high δ^15^N which are indicative of a population that is feeding on a similar trophic level^27^. Hence, at least in winter, females were feeding on enriched prey or on a higher trophic level.

On  the other hand, body size can affect isotopic composition if larger (winter) females capture prey of higher trophic position. Some evidence of a potential influence of body size on isotopic composition, irrespective of the season, is found in the positive and significant correlation between isotopic composition and female body size for the females collected in summer. Another line of evidence is the fact that prey size correlates with adult body size in *P. serratus*^49^. Intraspecific variation in ^15^N has been correlated to body size^50^. Such correlation may be due to ontogenetic variation in the diet^28,31^ or enrichment of the prey consumed. Matthews & Mazunder^51^ showed ^15^N to increase with size in both carnivorous and herbivorous zooplankton; such enlarged prey with enriched in δ^15^N may be more available to larger predators. Overall, both body size and season have the potential to mediate relationships between maternal nutrition and offspring traits in *P. serratus*.

In spite of the (expected) overall differences between female and offspring isotopic values, we found that their isotopic values correlated very closely: (1) both embryos and larvae from larger winter females reflect the higher isotopic values found in females; (2) when we restrict the analysis to the summer, the isotopic values of females, larvae and eggs correlated in the same manner with female body size. Eggs and females isotopic composition of *P. serratus* contrasted in δ^15^N by at least one “trophic level”. Bulk N isotope analysis may indicate modest trophic changes caused by relatively large trophic fractionation for many non-essential amino acids (AAs) and little to no fractionation for all essential AAs^52^. Essential AAs are more conserved across trophic levels because they retain approximately the same isotopic composition as dietary sources and thus specific AAs would have lower δ^15^N values, while non-essential AAs would be enriched^53^. The allocation of female resources to eggs in this marine invertebrate that result in lower δ^15^N in eggs may be due to the transfer of essential AAs^54^. These data suggest conservative patterns of fractionation or routing process from vitellogenesis to larval hatching where correlated seasonal-body size difference in egg and larval isotopic values may reflect differences in the proportion of essential amino acids or lipids; this hypothesis needs further evaluation.

Overall, the strong correlation between maternal and offspring size and isotopic composition found in *P. serratus*, highlights the need of research into examining links between maternal nutrition and offspring traits in order to better understand mechanisms underlying adaptive offspring trait variation. Our results may be relevant in cases where mothers produce offspring in different seasons (or habitats) and in cases where maternal size variation is important, for example influencing the size of captured preys^33,55–57^. An experimental approach is needed to determine if size-dependent maternal feeding behaviour can drive relationships between female size and offspring traits; or if alternatively seasonal variations in potential prey can drive variation in offspring traits. In investigating causes of offspring trait variation, stable isotopes can provide useful insights into effects of maternal diet and trophic level on offspring quality.

## Material and Methods

### Collection and maintenance of females

*Palaemon serratus* is a coastal decapod crustacean distributed across west European estuaries and coastal zones^58^. Embryos develop externally attached to the female pleopods and thus freshly hatched larvae emerge from the female after the embryogenesis. Twenty ovigerous females (size range 20.9–24.2 mm length of cephalothorax (Lc)) were obtained in January 2012 (winter) and another twenty (size range 14.0–18.2 mm cephalothorax length) in June 2012 (summer) from Isle of Anglesey, North Wales, UK.

In a first batch of females, we studied isotopic composition in muscle tissue of females and their corresponding early stage embryos. We focused on females with early stage embryos (noneyed stage) to avoid biases in isotopic composition produced by potential changes in metabolism of yolk reserves during embryogenesis. On the day of collection at each season, muscle tissue was sampled from ten females and two samples of eggs were preserved. A second batch of females were kept in the laboratory (80 days in winter and 28 days in summer) until larvae were released. These females were maintained individually at a temperature of 12 °C in the winter and 18 °C in the summer and a salinity of 32, and were fed mussels. We analysed isotopic composition of both eggs and freshly hatched larvae: two samples of embryos were separated from each female the same day of collection; at the time of hatching five samples of freshly hatched larvae were also collected. All samples were frozen at −30 °C and later freeze-dried. All methods in this study (using the invertebrate *Palaemon serratus*) were carried out in accordance with relevant guidelines and regulations within the School of Ocean Sciences, Menai Bridge, respecting the fundamental ethical principles of the UK, including those reflected in the Charter of Fundamental Rights of the European Union.

### Stable isotope analysis

Female muscle tissue was separated from other tissue in twenty adults (ten in January and ten in June 2012); eggs and larvae samples were obtained by pooling 30 eggs or 15 freshly hatched larvae. All samples were homogenised, weighed into tin cups (D1008, Elemental Microanalysis Ltd, UK) and analysed for carbon and nitrogen content and stable isotope ratios using a PDZ Europa Scientific Roboprep elemental analyser coupled to a PDZ Europa Hydra 20/20 stable isotope ratio mass spectrometer (Crewe, UK) at the Stable Isotope Facility, University of California, Davis. Stable isotope ratios in the samples are expressed as delta notation (δ, ‰), deviations from the isotopic ratios found in Pee Dee belemnite and atmospheric nitrogen. See Supplementary Information for the values of individual variation of the carbon and nitrogen isotopic values (‰) of the eggs, freshly hatched larvae and females.

### Data analysis

Data were analysed separately by batch, ie. (1) females and freshly spawned eggs; (2) eggs and freshly hatched larvae. We were interested in testing for effects of season, female size and ontogenetic variation (mother, egg, larva), but season and size co-varied to some extent. In order to tease apart effects of season vs. size we tested our hypotheses by fitting different statistical models. In “Model 1” we tested for the effect of season (summer and winter) and stage (mother, egg, larva) following a factorial design. In “Model 2” with tested for the maternal size, instead of season, and stage using an ANCOVA type model. In “Model 3”, we checked if season was significant after removing the effect of female size, using Type I sum of squares, considering size, season and mother-offspring effect. Finally, in “Model 4”, we tested for the effect of maternal size (and mother-offspring variation) within each season, in separate tests (model 4.1: winter; model 4.2: summer).

For δ^13^C and δ^15^N isotopic values we used PERMANOVA test (version 6, Primer-E Ltd., Plymouth, UK) as this was a multivariate response variable. We then run univariate analyses using generalized linear model GLM in R 3.2.5^59^.

### Data availability

Our data will be available at the Dyrad Data Archive (González-Ortegón *et al*. 2018) (http://datadryad.org).

## Electronic supplementary material

SUPPLEMENTARY TABLES AND FIGURES
