# Supplementary material for: Maternal Trophic Status and Offpsring Phenotype in a Marine Invertebrate
Source: Sci Rep. 2018 Jun 25;8:9618. doi: 10.1038/s41598-018-27709-2 (PMC6018471; doi:10.1038/s41598-018-27709-2)

SUPPLEMENTARY TABLES AND FIGURES

TITLE: MATERNAL TROPHIC STATUS AND OFFPSRING PHENOTYPES IN A MARINE INVERTEBRATE

Enrique González-Ortegón[12*], Lewis Le Vay[3], Mark Edward Mackay Walton[3] and Luis Giménez[3]

[1] ICMAN-Instituto de Ciencias Marinas de Andalucía (CSIC). Campus Universitario Río San Pedro, 11510 Puerto Real, Cádiz. Spain.

[2]CEI-MAR International Campus of Excellence of the Sea

[3] School of Ocean Sciences, Bangor University, Menai Bridge LL59 5AB, UK

quique.gonzalez@icman.csic.es, l.levay@bangor.ac.uk, m.walton@bangor.ac.uk and l.gimenez@bangor.ac.uk

*Corresponding author: quique.gonzalez@icman.csic.es

Supplementary Table S1. P values resulting from a GLM to evaluate the effects of season (summer vs. winter), stage (female-eggs and eggs-larvae) and body size on isotopic signal ($\delta^{13}C$ and $\delta^{15}N$), nutrient composition C:N and percentage of carbon and nitrogen of female muscle, eggs and larvae of *Palaemon serratus*. (Lc – female cephalothorax length). 2-way models: Model 1 (Season-Stage), Model 2 (Stage-Lc), Mode 4.1 (Lc-Stage in winter), Model 4.2 (Lc-Stage in summer); 3-way model: Model 3 (Season-Stage-Lc). Significant terms are highlighted in bold.

Females-Eggs								Eggs-Larvae					
Model 1	Source	df	$\delta^{13}C$	$\delta^{15}N$	C:N	C%	N%	df	$\delta^{13}C$	$\delta^{15}N$	C:N	C%	N%
	Season(Se)	1	**$<10^{-12}$**	**$<10^{-02}$**	**$<10^{-13}$**	**$<10^{-02}$**	**$<10^{-08}$**	1	**$<10^{-16}$**	**$<10^{-15}$**	**$<10^{-16}$**	**$<10^{-06}$**	**$<10^{-16}$**
	Stage(St)	1	**$<10^{-16}$**	**$<10^{-16}$**	**$<10^{-16}$**	**$<10^{-16}$**	**$<10^{-16}$**	1	**$<10^{-07}$**	**0.02**	**$<10^{-16}$**	**$<10^{-16}$**	**$<10^{-13}$**
	Se×St	1	0.10	**$<10^{-06}$**	**$<10^{-06}$**	**$<10^{-05}$**	0.08	1	0.92	0.89	0.05	0.05	**0.03**
	Res	55						112					
Model 2	Source	df	$\delta^{13}C$	$\delta^{15}N$	C:N	C%	N%	df	$\delta^{13}C$	$\delta^{15}N$	C:N	C%	N%
	Stage(St)	1	**$<10^{-16}$**	**$<10^{-16}$**	**$<10^{-16}$**	**$<10^{-16}$**	**$<10^{-16}$**	1	**$<10^{-3}$**	**$<10^{-05}$**	**$<10^{-16}$**	**$<10^{-16}$**	**$<10^{-07}$**
	Lc	1	**$<10^{-12}$**	**$<10^{-14}$**	**$<10^{-12}$**	**0.01**	**$<10^{-07}$**	1	**$<10^{-16}$**	**$<10^{-16}$**	**$<10^{-16}$**	**$<10^{-12}$**	**$<10^{-16}$**
	St×Lc	1	0.08	**$<10^{-06}$**	**$<10^{-06}$**	**$<10^{-05}$**	0.23	1	0.75	0.82	**0.04**	**0.04**	**0.01**
	Res	55						112					
Model 3	Source	df	$\delta^{13}C$	$\delta^{15}N$	C:N	C%	N%	df	$\delta^{13}C$	$\delta^{15}N$	C:N	C%	N%
	Stage(St)	1	**$<10^{-16}$**	**$<10^{-16}$**	**$<10^{-16}$**	**$<10^{-16}$**	**$<10^{-16}$**	1	**$<10^{-03}$**	**$<10^{-05}$**	**$<10^{-16}$**	**$<10^{-16}$**	**$<10^{-07}$**
	Season(Se)	1	**$<10^{-12}$**	**$<10^{-02}$**	**$<10^{-11}$**	**0.01**	**$<10^{-07}$**	1	**$<10^{-16}$**	**$<10^{-16}$**	**$<10^{-16}$**	**$<10^{-16}$**	**$<10^{-16}$**
	Lc	1	**0.04**	**$<10^{-02}$**	0.16	0.95	0.26	1	**$<10^{-03}$**	**$<10^{-10}$**	**$<10^{-03}$**	0.44	**$<10^{-06}$**
	St×Se	1	0.08	**$<10^{-06}$**	**$<10^{-06}$**	**$<10^{-05}$**	0.09	1	0.86	0.89	0.09	0.12	**0.04**
	St×Lc	1	0.93	0.80	0.13	0.84	0.24	1	0.62	0.49	0.33	**0.03**	0.13
	Se×Lc	1	0.11	0.29	0.82	0.46	0.74	1	**$<10^{-02}$**	**$<10^{-06}$**	**$<10^{-03}$**	**0.01**	**0.01**
	St×Se×Lc	1	0.63	0.64	0.76	0.81	0.49	1	0.92	0.95	0.25	**0.03**	0.14
	Res	51						108					
Model 4.1	Source	df	$\delta^{13}C$	$\delta^{15}N$	C:N	C%	N%	df	$\delta^{13}C$	$\delta^{15}N$	C:N	C%	N%
	Stage(St)	1	**$<10^{-16}$**	**$<10^{-14}$**	**$<10^{-16}$**	**$<10^{-16}$**	**$<10^{-16}$**	1	**$<10^{-11}$**	**0.01**	**$<10^{-16}$**	**$<10^{-16}$**	**$<10^{-06}$**
	Lc	1	0.70	0.37	0.09	0.61	0.63	1	0.86	0.44	0.48	**0.02**	0.06
	St×Lc	1	0.69	0.90	0.34	0.82	0.82	1	0.93	0.84	0.23	0.14	0.94
	Res	26						51					
Model 4.2	Source	df	$\delta^{13}C$	$\delta^{15}N$	C:N	C%	N%	df	$\delta^{13}C$	$\delta^{15}N$	C:N	C%	N%
Model 4.2	Stage(St)	1	**$<10^{-15}$**	**$<10^{-10}$**	**$<10^{-16}$**	**$<10^{-16}$**	**$<10^{-16}$**	1	**$<10^{-2}$**	0.07	**$<10^{-16}$**	**$<10^{-16}$**	**$<10^{-09}$**
Summer	Lc	1	**0.04**	**$<10^{-02}$**	0.42	0.54	0.27	1	**$<10^{-3}$**	**$<10^{-09}$**	**$<10^{-05}$**	**0.03**	**$<10^{-05}$**
	St×Lc	1	0.71	0.51	0.27	0.94	0.12	1	0.86	0.76	0.53	**0.04**	**0.03**
	Res	25						57					

Supplementary Table S2. Results of the 2-way and 3-way PERMANOVA for δ13C and δ15N in *Palaemon serratus*, design with factors Season (Summer and winter), Stage (Female, Larvae and eggs) and the covariate maternal cephalothorax length (Lc) as fixed factors. 2-way models: Model 1 (Season-Stage), Model 2 (Stage-Lc), Mode 4.1 (Lc-Stage in winter), Model 4.2 (Lc-Stage in summer); 3-way model: Model 3 (Season-Stage-Lc). The analysis is based on the modified Euclidean distance dissimilarity of tissues isotopic signal of δ13C and δ15N (99999 permutations). Significant terms are highlighted in bold.

	Females -Eggs					Eggs-Larvae			
Model 1	Source	df	SS	Pseudo-F	P(perm)	df	SS	Pseudo-F	P(perm)
	Season(Se)	1	23.467	47.134	**1e-5**	1	108.95	101.75	**1e-5**
	Stage(St)	1	343.02	688.96	**1e-5**	1	18.334	17.122	**1e-5**
	Se×St	1	6.5682	13.192	**2e-5**	1	2.42E-2	2.26E-2	0.9768
	Res	57	28.379			112	119.93		
Model 2	Source	df	SS	Pseudo-F	P(perm)	df	SS	Pseudo-F	P(perm)
	Stage(St)	1	341.06	804.32	**1e-5**	1	18.105	19.751	**1e-5**
	Lc	1	25.204	59.437	**1e-5**	1	126.41	137.9	**1e-5**
	St×Lc	1	5.1977	12.258	**1e-5**	1	6.2884E-2	6.86E-2	0.9338
	Res	55	23.322			112	102.67		
Model 3	Source	df	SS	Pseudo-F	P(perm)	df	SS	Pseudo-F	P(perm)
	Lc	1	25.204	60.474	**1e-5**	1	126.41	157.62	**1e-5**
	Season(Se)	1	0.2634	0.63199	0.5334	1	4.3266	5.3947	**0.0059**
	Stage(St)	1	341.45	819.28	**1e-5**	1	18.105	22.575	**1e-5**
	Lc×Se	1	0.8201	1.9678	0.1459	1	11.671	14.553	**2e-5**
	Lc×St	1	4.9896	11.972	**4e-5**	1	4.6788E-2	5.8339E-2	0.9427
	Se×St	1	0.71255	1.7097	0.1861	1	6.2716E-2	7.8198E-2	0.9229
	Lc×Se×St	1	9.4615E-2	0.22702	0.7984	1	4.8968E-3	6.1056E-3	0.9941
	Res	51	21.255			108	86.617		
Model 4.1	Source	df	SS	Pseudo-F	P(perm)	df	SS	Pseudo-F	P(perm)
	Lc	1	0.18999	0.64336	0.502	1	0.122	0.43415	0.5505
	Stage(St)	1	206.61	699.62	**1e-5**	1	7.3339	26.098	**1e-5**
	Lc×St	1	1.5329E-2	5.1907E-2	0.9453	1	7.9105E-3	2.815E-2	0.9523
	Res	26	7.6782			51	14.332		
Model 4.2	Source	df	SS	Pseudo-F	P(perm)	df	SS	Pseudo-F	P(perm)
	Lc	1	4.5278	6.9845	**0.0039**	1	33.284	26.621	**1e-5**
	Stage	1	141.4	218.12	**1e-5**	1	11.428	9.1398	**0.0008**
	Lc×St	1	4.5672E-2	7.0454E-2	0.9284	1	7.0698E-2	5.6545E-2	0.942
	Res	27	17.503			58	72.518		

Supplementary Table S3. Average and coefficient of variation in δ13C and δ15N of females, larvae and eggs of *Palaemon serratus* in summer and winter samples.

Isotopes	$\delta^{13}C$						$\delta^{15}N$					
Stages	Female		Larvae		Eggs		Female		Larvae		Eggs	
	Mean	CV	Mean	CV	Mean	CV	Mean	CV	Mean	CV	Mean	CV
Summer	-17.08	3.4	-20.11	4.6	-21.25	4.5	14.76	2.7	13.07	6.5	13.04	5.8
Winter	-17.90	1.9	-21.76	1.3	-22.74	1.1	15.95	2.5	14.22	3.0	13.44	4.0

Supplementary Figure S1. Individual variation of the carbon and nitrogen content from female to eggs and from eggs to freshly hatched larvae sampled over 2 seasons (winter vs. summer).

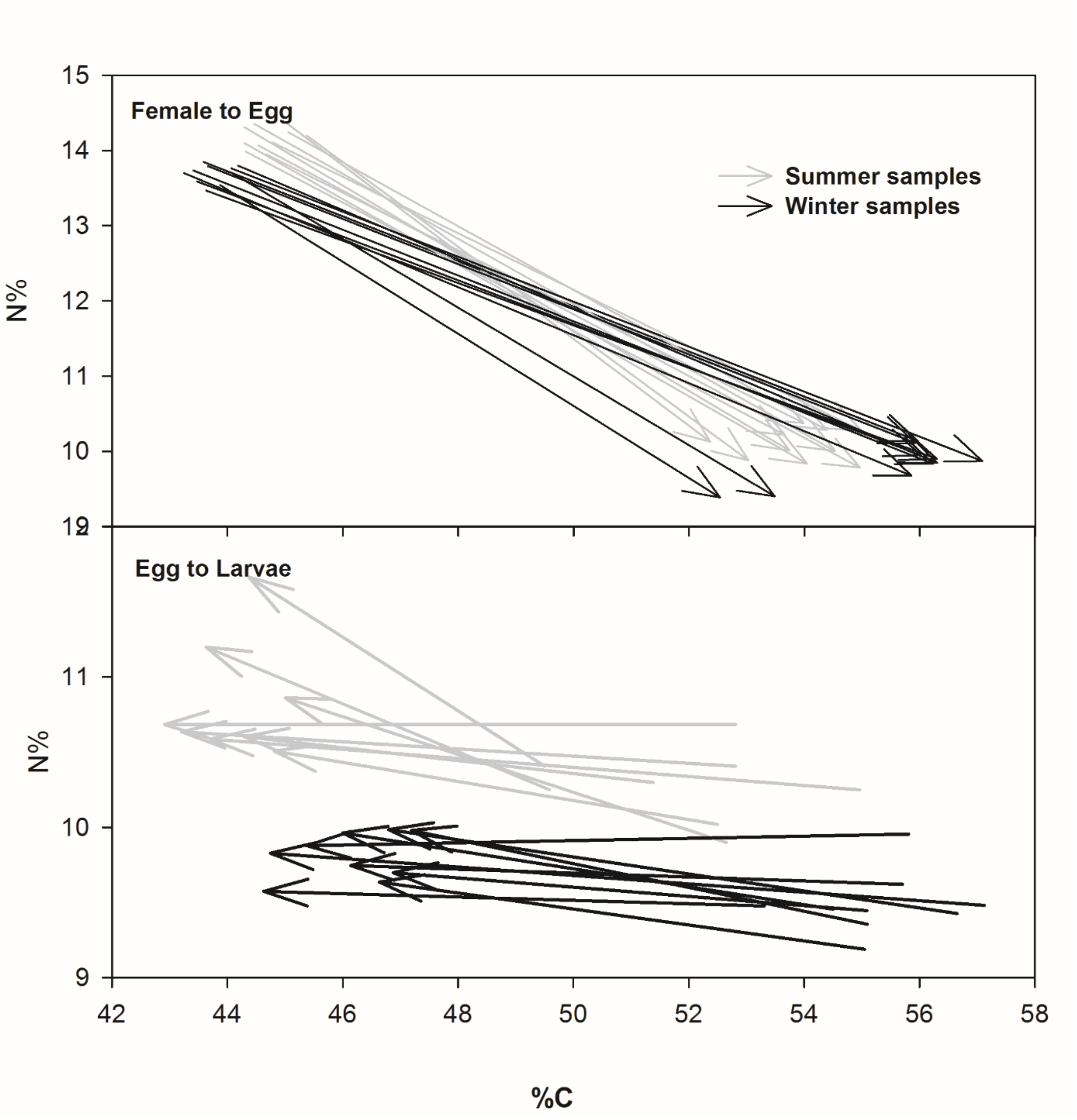

Supplementary Figure S2. Individual variation of the carbon and nitrogen isotopic signal (‰) from female to eggs and from eggs to freshly hatched larvae sampled over 2 seasons (winter vs. summer).

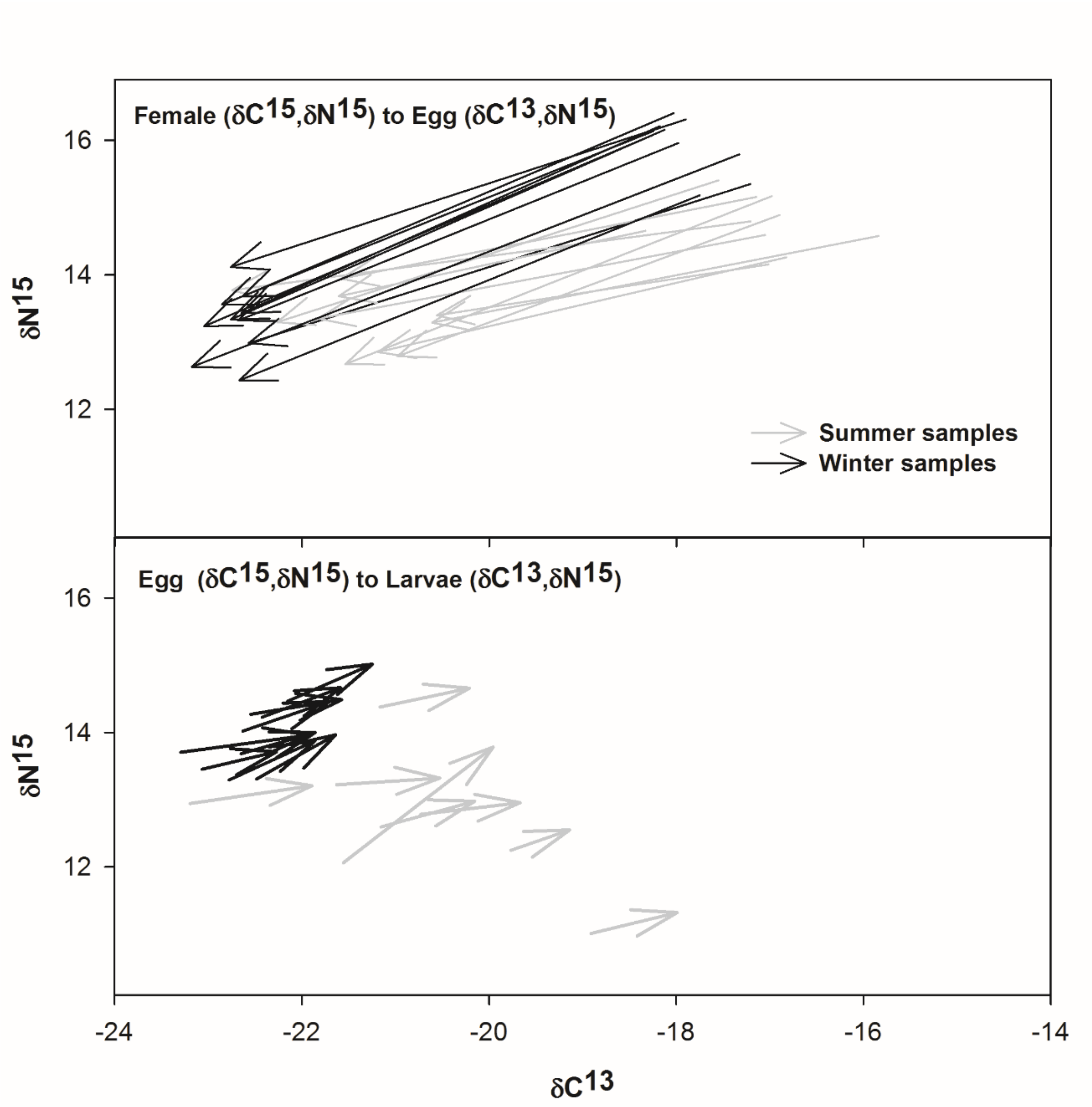

Supplement: Supplementary file 1 — SUPPLEMENTARY TABLES AND FIGURES [file 41598_2018_27709_MOESM1_ESM.pdf]
